# Dengue vaccines: what we know, what has been done, but what does the future hold?

**DOI:** 10.1590/S0034-8910.2015049006146

**Published:** 2015-09-29

**Authors:** Emiliana Pereira Abrão, Danillo Lucas Alves Espósito, Flávio Lauretti, Benedito Antonio Lopes da Fonseca

**Affiliations:** Departamento de Clínica Médica. Faculdade de Medicina de Ribeirão Preto. Universidade de São Paulo. Ribeirão Preto, SP, Brasil

**Keywords:** Dengue Vaccines, Dengue, prevention & control, Scientific Research and Technological Development, Communicable Disease Control

## Abstract

Dengue, a disease caused by any of the four serotypes of dengue viruses, is the most important arthropod-borne viral disease in the world in terms of both morbidity and mortality. The infection by these viruses induces a plethora of clinical manifestations ranging from asymptomatic infections to severe diseases with involvement of several organs. Severe forms of the disease are more frequent in secondary infections by distinct serotypes and, consequently, a dengue vaccine must be tetravalent. Although several approaches have been used on the vaccine development, no vaccine is available against these viruses, especially because of problems on the development of a tetravalent vaccine. Here, we describe briefly the vaccine candidates available and their ability to elicit a protective immune response. We also discuss the problems and possibilities of any of the vaccines in final development stage reaching the market for human use.

## Abstract (pt)

Dengue, doença causada por qualquer um dos quatro sorotipos dos vírus dengue, é atualmente a mais importante doença viral transmitida por artrópodos em todo o mundo, tanto em termos de morbidade como de mortalidade. A infecção por estes vírus causa grande variedade de manifestações clínicas, desde infecções assintomáticas até doenças graves com envolvimento de diversos órgãos. As formas graves da dengue são mais frequentes em infecções secundárias por sorotipos diferentes e, por esta razão, a vacina contra a dengue deve ser tetravalente. Embora várias estratégias tenham sido usadas no desenvolvimento de vacinas contra a dengue, não há ainda nenhuma vacina disponível, particularmente por problemas no desenvolvimento de uma vacina tetravalente. Aqui, descreve-se brevemente os candidatos vacinais disponíveis e a capacidade de eles induzirem resposta imune protetora contra novas infecções. Ainda, discutimos os problemas e as possibilidades de liberação, para uso em seres humanos, de qualquer uma das vacinas em fase final de desenvolvimento

## INTRODUCTION

Dengue is an acute febrile illness caused by the infection with any of dengue viruses. These arthropod-borne viruses belong to the genus *Flavivirus* from the family Flaviviridae, and are serologically classified into four distinct serotypes, dengue-1 to -4 (DENV-1 to DENV-4). These viruses are transmitted to humans by the bite of infected females of *Aedes *mosquitos, mainly by *Aedes aegypti*,^35^ and generate a lifelong protective immune response against homologous dengue strains and transient cross-protection against the other serotypes for a few months after the primary infection.^35^

Dengue viruses cause an important and rapidly spreading arboviral disease and are widely distributed in tropical and subtropical regions of the world.^2^ Dengue is endemic in more than 100 countries including Southeast Asia, South and Central America, the Caribbean and South Pacific regions and, among them, more than 60 countries have now reported dengue hemorrhagic fever (DHF).^35^ The World Health Organization (WHO) estimates that 50 to 100 million dengue infections occur annually and that approximately 2.5 billion people live in endemic areas where 500,000 to 1,000,000 dengue infections evolve to DHF/dengue shock syndrome (DSS), resulting in approximately 25,000 deaths.^35^ However, these numbers could be underestimated, and this hypothesis has been corroborated by a recent study by Bhatt et al,^2^ who used a formal modeling framework and suggest that approximately 390 million humans are infected every year, more than three times the previously reported by WHO.

A considerable portion of dengue virus infections result in asymptomatic infections, but also in clinical manifestations that include mild undifferentiated fever, dengue fever, and many more severe disease manifestations, the most studied one being DHF/DSS, in which the increased vascular permeability is the hallmark.^35^

Up to now, no animal model has allowed to conclude which are the precise mechanisms involved in the pathogenesis of dengue virus infection. Therefore, the pathophysiologic processes on the causation of severe dengue infections are not completely understood in spite of several studies that have addressed this issue. Several hypotheses that include mechanisms involving both viral and host factors have been proposed, such as the virulence of different virus strains,^24^ host genetics,^5^ ^,^ ^27^ the multifactorial interaction,^17^ the immune-mediated mechanisms,^4^ ^,^ ^8^ and the antibody-dependent enhancement.^14^ The latter phenomenon, mainly observed in epidemiological studies, may have an important role in the pathogenesis of DHF/DSS and is related to an enhancement of the disease severity after a secondary infection. The non-neutralizing heterotypic antibodies from a first infection enhance the viral entry into Fc gamma receptor (FcγR)-bearing cells, thus enhancing the viral load and consequently increasing the immune response.^13^ ^,^ ^29^

Currently, there is no specific drug for dengue treatment, and mosquito control is mandatory to contain the disease in affected countries. In this context, a preventive dengue vaccine is essential to control the disease and has been a priority for the WHO for several decades. The challenges to vaccine development lay on the complex immunopathogenesis of the disease, especially the antibody-dependent enhancement of infection phenomenon, requiring the development of tetravalent vaccines. These vaccines should provide long-term protection against all virus serotypes, an essential requirement to vaccine approval by regulatory agencies.^35^ Dengue vaccine research ranges from traditional methods of virus inactivation to advanced molecular biology strategies. Here we discuss these strategies briefly and point the problems with each one and the perspectives for a licensed dengue vaccine.

### Dengue vaccine candidates under development and on clinical trials

#### DNA vaccines

In recent decades, DNA vaccination has been used to induce cellular immune responses against various antigens, using *in vitro* or animal models, some of them being evaluated on clinical trials. This technology is based on cloning a specific fragment or gene into a bacterial plasmid containing a strong promoter for optimal expression in mammalian cells. Several preclinical DNA vaccine candidates were engineered against DENV but, up to this present publication, only two have been approved to be tested in humans.

A monovalent plasmid DNA vaccine against DENV-1 (D1ME^100^) that encodes the premembrane (prM) and envelope (E) proteins was tested in 22 healthy adults by intramuscular injection. Phase 1 trial results showed this vaccine candidate to be safe, but only five subjects that received higher doses (5 mg) had detectable neutralizing antibody responses (about 20.0%). An increase in IFN-γ expression and DENV-1 serotype cross-reactivity immune responses to DENV-2, -3 and -4 were also reported.^1^

Another DNA vaccine candidate approved for phase I of clinical trial, produced by the U.S. Naval Medical Research Center and Vical Incorporated, is a Tetravalent DENV Vaccine (TVDV) that also encodes prM and E genes, but from all serotypes (1-4), in a backbone plasmid VR1012 (Vical Incorporated). Preclinical studies immunizing Indian rhesus monkeys with this tetravalent vaccine associated with Vaxfectin^®^ adjuvant (Vical Incorporated) showed lower viremia in animals inoculated with the TVDV + Vaxfectin^®^ when challenged with live DENV-2 than control animals. The authors also reported a 2- to 10-fold increase in the neutralizing antibody response against DENV-1, -3 and -4.^22^

Although DNA vaccines seem to be an excellent approach to the development of dengue vaccines, the tests with these vaccine candidates are in their infancy, compared with other strategies.

#### Live attenuated and inactivated virus vaccines

Live attenuated virus vaccines have been successfully developed for other viruses and this strategy has also been used to develop a dengue vaccine candidate. These vaccines, made of weakened viruses, are excellent immunogens because they can induce humoral and cellular immune responses similar to a natural infection, but viral replication is insufficient to produce disease. The attenuated vaccine for yellow fever virus, another virus from the genus *Flavivirus*, is one of the most effective available in the market.^23^ The production of an attenuated vaccine against dengue virus was not as successful as for yellow fever virus. The Walter Reed Army Institute of Research (WRAIR) and GlaxoSmithKline (GSK) have infected primary dog kidney (PDK) cells with all four DENV serotypes to produce a tetravalent DENV vaccine expecting these vaccines to produce low viremia in recipients.^33^ The vaccine, which is in clinical phase II, was tested in flavivirus-naive Thai adults and showed to be safe and immunogenic. After two immunization doses, almost all subjects became seropositive to all DENV types.^33^ This approach, tested in adults and infants (by WRAIR and Mahidol University, respectively), did not show the same seroconversion success.^28^ ^,^ ^32^

On the other hand, inactivated vaccines are made from viruses that become noninfectious by heating or formaldehyde inactivation. They are safer than attenuated virus candidates because of the inability of reversion to virulence and do not represent any harm to immunocompromised individuals. A tetravalent dengue inactivated vaccine (DPIV), developed by Fiocruz (Brazil) in collaboration with GSK and WRAIR, is on phase I clinical trial, and showed to be protective and immunogenic in nonhuman primates in a preclinical test.^11^

#### Recombinant protein vaccines

Recombinant subunit vaccines are widely developed for several pathogens because of their safety profile, but they do not produce the same immunological responses to pathogens as live attenuated vaccines since they contain only one or few viral proteins. The DENV envelope protein is the most immunogenic and generally used for vaccine production.^7^ Hawaii Biotech/Merck developed a subunit vaccine candidate against all dengue serotypes containing a truncated E protein (DEN-80E) expressed in Drosophila S2 cells.^9^ Preclinical results showed an induction of high-titer neutralizing antibodies in both mice and nonhuman primates when administered with a specific adjuvant (ISCOMATRIX^TM ^– CSL).^9^ This vaccine is currently in phase I clinical trial in healthy young Australian adults, and three doses will be administered at monthly intervals.

#### The chimeric dengue virus vaccines

The yellow fever/dengue virus (CYD) vaccine is the one in the most advanced stage on dengue vaccine development, being evaluated on large-scale clinical trials around the world and tested in more than 10,000 children in five Southeast Asian countries and in more than 20,000 subjects in five countries in Latin America, including Brazil.^20^ This vaccine candidate is based on a yellow fever virus backbone (vaccine strain 17D) with the yellow fever virus prM and E genes replaced by DENV type-specific prM and E genes.

The results of the phase IIb clinical trial that took place in Thailand were released recently.^26^ In this study, 4,002 healthy Thai schoolchildren aged 4 to 11 years were randomly assigned to receive three injections of dengue vaccine or control (rabies vaccine or placebo) at zero, six and 12 months to assess its protective efficacy against virologically confirmed, symptomatic dengue occurring one month or longer after the third dose. Dengue disease occurred in 134 children during the study. Overall efficacy for all four serotypes was 30.2%, but only 9.0% for DENV-2, although neutralizing antibodies, measured shortly after the last doses, had relatively high mean titers.

The most recent results, obtained in a phase III study carried out in the Asia-Pacific region that evaluated 10,275 healthy children aged 2 to 14 years, were consistent with the phase IIb in Thailand, and the overall efficacy was 56.5%, but only 35.0% for DENV-2.^6^ Perhaps the best news of this vaccine was a decrease in cases of dengue hemorrhagic fever, the most severe form of the disease, by 88.5%. Of greater concern is the relative vaccine inefficacy in dengue virus-naive participants (35.5%), suggesting that this vaccine boosts and broadens preexisting immunity rather than primarily raising protective immunity, which might also explain the better efficacy in older children exposed to the virus.^34^ Another issue of concern is the lower efficacy against DENV-2 disease, although it is hard to explain why the presence of specific neutralizing antibodies is unprotective against dengue. Viral interference poses a probable problem in live tetravalent formulations resulting in an unequal immune response to each of the four serotypes.^15^

It had already been observed in early preclinical studies with CYD vaccine in monkeys that the simultaneous administration of tetravalent formulations produces a hierarchy of immune responses, with DENV-4 showing the best and DENV-2 the worst ones.^12^ Also, the concept that the infection with one serotype will confer lifelong protection to subsequent infections against serotypes from different regions of the world is under scrutiny. Most cohort studies on this concept were done in areas where each serotype is represented by a single circulating genotype and the role of genotypic variation of a serotype in repeated infections is less certain.^31^

Sabchareon et al^26^ hypothesized that the poor level of protection of CYD vaccine could be due to a mismatch between the vaccine strain and the genetically divergent circulating DENV-2 Asian strain. Studies with mouse and human convalescent sera show that the neutralizing activity against DENV-3 from different genotypes of the same serotype varies in the ability to neutralize the virus infection *in vitro.* ^19^ ^,^ ^25^ ^,^ ^30^ If true, this hypothesis has an enormous impact in future dengue vaccines, because instead of a universal vaccine, each vaccine candidate will have to be designed to specific circulating strains around the world.

Lastly, the lack of DENV T-cell immunity in CYD vaccine is the most likely reason for the lack of multitypic protective immunity.^15^ Data from mouse models and recent studies in humans suggest that T-cell immunity contributes importantly to protection against infection by different serotypes of DENV. In this regard, the role of nonstructural proteins (NS) in eliciting cellular immunity is being considered in dengue vaccine approaches. Studies with DENV-infected patients or volunteers receiving candidate vaccines evidenced CD4+ and CD8+ T-cell epitopes throughout NS3.^18^ It is plausible that vaccines presenting yellow fever virus but not DENV nonstructural protein antigens, such as the CYD vaccine, do not mount a strong and protective anti-DENV T-cell response.^15^

#### The chimeric live attenuated DENV-2/DENV vaccine (DENVax)

The DENVax was constructed using the backbone of the cell culture attenuated DENV-2 (PDK-53 strain), in which the prM and E genes of DENV-2 PDK-53 were substituted with those of wild-type DENV-1, -3, or -4.^16^ DENVax is undergoing extensive safety and tolerability tests on phase I and II clinical trials in Colombia, Puerto Rico, Singapore and Thailand. In Colombia, 96 healthy individuals aged 18 to 45 years were assigned to receive two doses of tetravalent DENVax 90 days apart. Results show that the vaccine was well tolerated and immunogenic. Thirty days after a second dose, 62.0% of patients seroconverted to all four dengue serotypes.^21^ The next steps will concentrate in the efficacy trials. Although promising results are expected, the lessons learned with Chimerivax vaccine draw our attention to the fact that protection against dengue disease should not rely solely in humoral immunity but also in the cellular immune responses. Thus, additional markers of protection such as T-cell activation, interferon-gamma (INF-g) and tumor necrosis factor-alpha (TNF-a) production should be used on immunogenicity assays.^15^

#### The chimeric live attenuated DENV/DENV vaccine (TetraVax-DV)

Another approach was used to produce a different tetravalent DENV vaccine (TetraVax-DV vaccine). Attenuation was achieved by a deletion of 30 nucleotides (Δ30) on 3’ untranslated region of wild-type DENV-1 and -4. Since this approach did not result in attenuation for the other two viruses, DENV-2 chimeric vaccine candidate was generated by replacing prM and E genes on the DEN4Δ30 backbone with those of the wild-type viruses and DENV-3 vaccine candidate by additional deletion of the 3’ untranslated region, respectively. These vaccine candidates retain wild-type structural proteins to maximize infectivity, thereby decreasing the potential for virus interference, and also to increase the magnitude and extension of the neutralizing antibody response.^3^ ^,^ ^7^

TetraVax has been tested for safety and immunogenicity in flavivirus-naive subjects in the United States. All vaccines were safe and immunogenic and one formulation was shown to induce tetravalent immune response in 79.0% of subjects after only a single dose.^7^ ^,^ ^10^ The vaccine is being tested for its safety and immunogenicity profile in phase II Clinical Trial in several sites around the world.

## CONCLUSION

As mentioned above, several candidate vaccines are undergoing clinical trials. Most of them elicit good profiles of immunogenicity and reactogenicity when administered as a single vaccine, but none have achieved a good percentage of protection against dengue infections when used as a tetravalent vaccine. The reasons for this low efficacy are the possible viral interference among virus strains combined in the vaccines, the uncertainty about the levels of protective titers of neutralizing antibodies, the possible lack of protection against strains different from those contained in the vaccines, and the evidence for the need of nonstructural proteins to elicit an adequate immune response. All candidate vaccines will have to deal with these questions before they are approved for human use. Finally, the vaccine candidate in the most advanced stage of development has just completed a phase III clinical trial and has induced an efficacy of a little less than 60.0%, and this efficacy has been achieved only after three doses of the vaccine. Even though this efficacy would result in a considerable reduction in the number of dengue cases, the questions are concerned to whom is going to use this vaccine and if there will be vaccines available for the whole world, in particular for developing countries, where dengue represents an important public health problem.
